# Bis(1,10-phenanthroline-κ^2^ *N*,*N*′)(sulfato-κ*O*)zinc(II) propane-1,2-diol monosolvate

**DOI:** 10.1107/S160053681302610X

**Published:** 2013-09-28

**Authors:** Kai-Long Zhong

**Affiliations:** a Department of Applied Chemistry, Nanjing College of Chemical Technology, Nanjing, 210048, People’s Republic of China

## Abstract

In the title compound, [Zn(SO_4_)(C_12_H_8_N_2_)_2_]·C_3_H_8_O_2_, the Zn^II^ ion is in a distorted square-pyramidal coordination environment composed of four N atoms from two chelating 1,10-phenanthroline ligands and one O atom from a monodentate sulfate ligand. The Zn^II^ ion lies on a twofold rotation axis. The sulfate ligand and propane-1,2-diol mol­ecules are disordered across the twofold rotation axis. The dihedral angle between the two chelating N_2_C_2_ groups is 83.26 (13)°. In the crystal, the complex mol­ecule and the propane-1,2-diol mol­ecule are connected through a pair of O—H⋯O hydrogen bonds.

## Related literature

For the ethane-1,2-diol solvate of the title complex, see: Zhu *et al.* (2006 ▶) and for the propane-1,3-diol solvate of the title complex, see: Cui *et al.* (2010 ▶). For related structures and background references, see: Batten & Robson (1998 ▶); Zhang *et al.* (2010 ▶); Zhong (2010 ▶); Zhong *et al.* (2011 ▶).
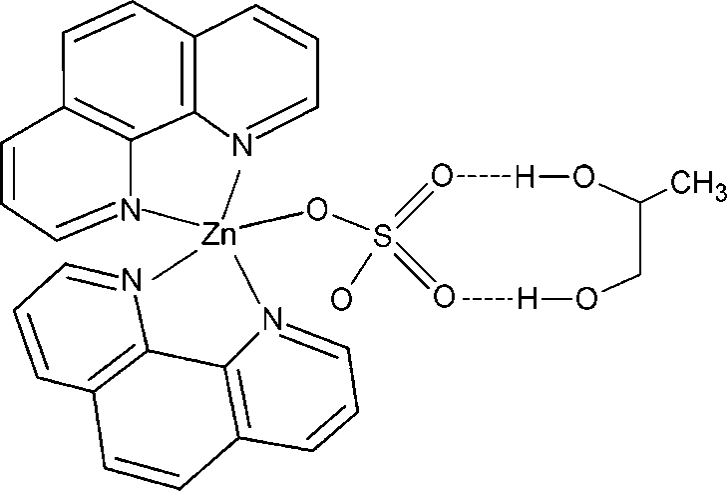


## Experimental

### 

#### Crystal data

[Zn(SO_4_)(C_12_H_8_N_2_)_2_]·C_3_H_8_O_2_
*M* *_r_* = 597.93
Monoclinic, 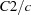

*a* = 17.3913 (10) Å
*b* = 12.9247 (7) Å
*c* = 13.2214 (7) Å
β = 123.248 (5)°
*V* = 2485.4 (2) Å^3^
*Z* = 4
Mo *K*α radiation
μ = 1.13 mm^−1^
*T* = 223 K
0.35 × 0.20 × 0.15 mm

#### Data collection

Rigaku Mercury CCD diffractometer
Absorption correction: multi-scan (*REQAB*; Jacobson, 1998 ▶) *T*_min_ = 0.968, *T*_max_ = 1.000
5735 measured reflections
2191 independent reflections
1878 reflections with *I* > 2σ(*I*)
*R*_int_ = 0.035

#### Refinement

*R*[*F* ^2^ > 2σ(*F* ^2^)] = 0.041
*wR*(*F* ^2^) = 0.102
*S* = 1.06
2191 reflections
186 parameters
4 restraints
H-atom parameters constrained
Δρ_max_ = 0.73 e Å^−3^
Δρ_min_ = −0.54 e Å^−3^

### 

Data collection: *CrystalClear* (Rigaku, 2007 ▶); cell refinement: *CrystalClear*; data reduction: *CrystalClear*; program(s) used to solve structure: *SHELXS97* (Sheldrick, 2008 ▶); program(s) used to refine structure: *SHELXL97* (Sheldrick, 2008 ▶); molecular graphics: *XP* in *SHELXTL* (Sheldrick, 2008 ▶); software used to prepare material for publication: *SHELXTL*.

## Supplementary Material

Crystal structure: contains datablock(s) global, I. DOI: 10.1107/S160053681302610X/lh5651sup1.cif

Structure factors: contains datablock(s) I. DOI: 10.1107/S160053681302610X/lh5651Isup2.hkl

Additional supplementary materials:  crystallographic information; 3D view; checkCIF report

## Figures and Tables

**Table 1:** Hydrogen-bond geometry (Å, °)

*D*—H⋯*A*	*D*—H	H⋯*A*	*D*⋯*A*	*D*—H⋯*A*
O5—H5⋯O3	0.83	2.00	2.74 (3)	149
O6—H6⋯O4	0.83	2.11	2.89 (2)	155

**Fig. 1.**
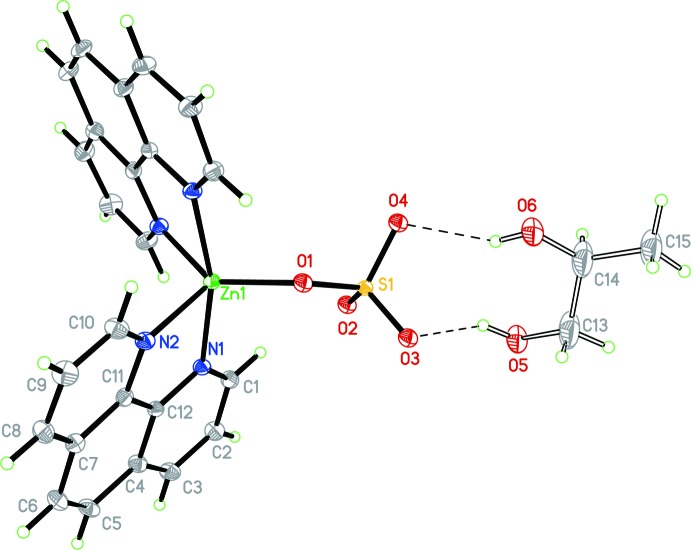
Figure 1. The molecular structure showing displacement ellipsoids drawn at the 30% probability level. The light broken lines depict O—H···O hydrogen bonds. The propane-1,2-diol molecule and the sulfate ligand are disordered over two symmetry-related positions but the disorder is not shown. Unlabeled atoms are related to the labeled atoms by the symmetry operator (-x, y, -z + 1/2).

## supplementary crystallographic information

### 1. Comment

The design and synthesis of metal-organic complexes or polymeric coordination networks is a rapidly developing field in coordination and supramolecular chemistry during the past decades (Batten & Robson, 1998; Zhang *et al.*, 2010; Zhong *et al.*, 2011). In our inverstigation, we have focused on the synthesis of complexes with N containing bidentate ligands, such as 1,10-phenanthroline (phen), 4,4'-bipyridine and 2,2'-bipyridine as auxiliary ligands, meanwhile retaining some of the solvent molcecules capable of hydrogen bonding to form higher dimensional supramolecular network. In the past few years, we have synthesized and reported Zn-complexes with bidentate-chelating sulfate ions, in which uncoordinated O atoms of the sulfate ligand and dihydric alcohol solvent molecules formed classical O—H···O hydrogen bonds *vis* a solvothermal reaction, *e.g.* [ZnSO_4_(phen)_2_]·C_2_H_6_O_2_, (II), (C_2_H_6_O_2_ is ethane-1,2-diol; Zhu *et al.*, 2006). [ZnSO_4_(phen)_2_]·C_3_H_8_O_2_, (III), (C_3_H_8_O_2_ is propane-1,3-diol; Cui *et al.*, 2010) and [ZnSO_4_(2,2'-bipy)_2_]·C_2_H_6_O_2_ (Cui *et al.*, 2010). The crystal structure of the title complex is reported herein.

The title compound consists of a neutral monomeric [ZnSO_4_(C_10_H_8_N_2_)_2_] complex and a propane-1,2-diol solvent molecule. The Zn^II^ ion has fivefold coordination by four N atoms from two phen ligands and one O atom from an monodentate sulfate ion, in a distorted ZnN_4_O square-pyramidal environment. This is different from that observed in previously reported zinc complexes (II) and (III). The Zn—N bond distances, the Zn—O bond distance, the N—Zn—N bite angle and the dihedral angle between the two chelating NCCN groups are 2.123 (3)–2.142 (2) Å, 1.959 (4) Å, 78.32 (9)° and 83.26 (13)°, respectively. The Zn^II^ ion is located on a twofold rotation axis (symmetry code: -*x*, *y*, - *z* + 1/2)(Fig.1). The sulfate ligand and propane-1,2-diol molecules are disordered across the twofold rotation axis. Depending on the symmetry unique component of disorder, either N2 or N2 (-x, y, -z+1/2) forms the apical atom of the disordered square-pyramidal coordination geometry. The [ZnSO_4_(C_10_H_8_N_2_)_2_] and C_3_H_8_O_3_ units are connected by a pair of intermolecular O—H···O hydrogen bonds involving the uncoordinated O atoms of the sulfate ligand.

### 2. Experimental

0.2 mmol phen, 0.1 mmol melamine, 0.1 mmol ZnSO_4_·7H_2_O, 2.0 ml propane-1,2-diol and 1.0 ml water were mixed and placed in a thick Pyrex tube, which was sealed and heated to 453 K for 72 h, whereupon colorless block-shaped crystals of (I) were obtained.

### 3. Refinement

All non-hydrogen atoms were refined anisotropically. The H atoms of phen were positioned geometrically and allowed to ride on their parent atoms, with C—H = 0.93 Å and *U*_iso_(H) = 1.2*U*_eq_(C). The H atoms of propane-1,2-diol were placed in geometrically idealized positions and refined as riding atoms, with C—H(CH_3_) = 0.96 Å, C—H(CH_2_) = 0.97 Å C—H(CH) = 0.98 Å and O—H = 0.82 Å; *U*_iso_(H) = 1.2*U*_eq_(C) and 1.5*U*_eq_(O).

The solvent molecule propane-1,2-diol and the sulfate ion are disordered over the twofold rotation axis and the unique sites were refined with 0.50 site occupancy.

### Crystal data

**Table:**

[Zn(SO_4_)(C_12_H_8_N_2_)_2_]·C_3_H_8_O_2_	*F*(000) = 1232
*M**_r_* = 597.93	*D*_x_ = 1.598 Mg m^−^^3^
Monoclinic, *C*2/*c*	Mo *K*α radiation, λ = 0.71073 Å
Hall symbol: -C 2yc	Cell parameters from 2296 reflections
*a* = 17.3913 (10) Å	θ = 3.5–28.8°
*b* = 12.9247 (7) Å	µ = 1.13 mm^−^^1^
*c* = 13.2214 (7) Å	*T* = 223 K
β = 123.248 (5)°	Block, colourless
*V* = 2485.4 (2) Å^3^	0.35 × 0.20 × 0.15 mm
*Z* = 4	

### Data collection

**Table:**

Rigaku Mercury CCD diffractometer	2191 independent reflections
Radiation source: fine-focus sealed tube	1878 reflections with *I* > 2σ(*I*)
Graphite Monochromator monochromator	*R*_int_ = 0.035
Detector resolution: 28.5714 pixels mm^-1^	θ_max_ = 25.0°, θ_min_ = 2.8°
ω scans	*h* = −20→20
Absorption correction: multi-scan (REQAB; Jacobson, 1998)	*k* = −15→14
*T*_min_ = 0.968, *T*_max_ = 1.000	*l* = −14→15
5735 measured reflections	

### Refinement

**Table:**

Refinement on *F*^2^	Primary atom site location: structure-invariant direct methods
Least-squares matrix: full	Secondary atom site location: difference Fourier map
*R*[*F*^2^ > 2σ(*F*^2^)] = 0.041	Hydrogen site location: inferred from neighbouring sites
*wR*(*F*^2^) = 0.102	H-atom parameters constrained
*S* = 1.06	*w* = 1/[σ^2^(*F*_o_^2^) + (0.0486*P*)^2^ + 2.9011*P*] where *P* = (*F*_o_^2^ + 2*F*_c_^2^)/3
2191 reflections	(Δ/σ)_max_ < 0.001
186 parameters	Δρ_max_ = 0.73 e Å^−^^3^
4 restraints	Δρ_min_ = −0.54 e Å^−^^3^

### Special details

**Table:**

Geometry. All e.s.d.'s (except the e.s.d. in the dihedral angle between two l.s. planes) are estimated using the full covariance matrix. The cell e.s.d.'s are taken into account individually in the estimation of e.s.d.'s in distances, angles and torsion angles; correlations between e.s.d.'s in cell parameters are only used when they are defined by crystal symmetry. An approximate (isotropic) treatment of cell e.s.d.'s is used for estimating e.s.d.'s involving l.s. planes.
Refinement. Refinement of *F*^2^ against ALL reflections. The weighted *R*-factor *wR* and goodness of fit *S* are based on *F*^2^, conventional *R*-factors *R* are based on *F*, with *F* set to zero for negative *F*^2^. The threshold expression of *F*^2^ > σ(*F*^2^) is used only for calculating *R*-factors(gt) *etc*. and is not relevant to the choice of reflections for refinement. *R*-factors based on *F*^2^ are statistically about twice as large as those based on *F*, and *R*- factors based on ALL data will be even larger.

### Fractional atomic coordinates and isotropic or equivalent isotropic displacement parameters (Å^2^)

**Table:**

	*x*	*y*	*z*	*U*_iso_*/*U*_eq_	Occ. (<1)
Zn1	0.0000	0.30049 (4)	0.2500	0.02159 (19)	
S1	0.0034 (2)	0.54103 (11)	0.2316 (3)	0.0167 (6)	0.50
O1	0.0136 (3)	0.4449 (3)	0.3022 (4)	0.0257 (5)	0.50
O2	−0.0007 (3)	0.5122 (3)	0.1220 (4)	0.0257 (5)	0.50
O3	0.0811 (6)	0.6077 (9)	0.3077 (9)	0.0257 (5)	0.50
O4	−0.0835 (7)	0.5935 (8)	0.1998 (10)	0.0257 (5)	0.50
O5	0.0562 (14)	0.7871 (13)	0.1824 (16)	0.046 (2)	0.50
H5	0.0423	0.7309	0.1988	0.069*	0.50
O6	−0.0460 (14)	0.7974 (14)	0.3059 (16)	0.046 (2)	0.50
H6	−0.0423	0.7429	0.2756	0.069*	0.50
N1	0.09382 (15)	0.27777 (19)	0.1947 (2)	0.0222 (6)	
N2	0.10269 (16)	0.2072 (2)	0.3926 (2)	0.0228 (6)	
C1	0.0913 (2)	0.3167 (2)	0.1003 (3)	0.0261 (7)	
H1A	0.0459	0.3660	0.0520	0.031*	
C2	0.1535 (2)	0.2875 (3)	0.0694 (3)	0.0284 (7)	
H2A	0.1497	0.3170	0.0018	0.034*	
C3	0.2194 (2)	0.2159 (3)	0.1382 (3)	0.0297 (8)	
H3A	0.2607	0.1947	0.1173	0.036*	
C4	0.2257 (2)	0.1735 (2)	0.2406 (3)	0.0248 (7)	
C5	0.2950 (2)	0.1021 (3)	0.3207 (3)	0.0284 (7)	
H5A	0.3380	0.0786	0.3038	0.034*	
C6	0.3001 (2)	0.0674 (3)	0.4204 (3)	0.0293 (8)	
H6A	0.3465	0.0202	0.4719	0.035*	
C7	0.2354 (2)	0.1019 (2)	0.4489 (3)	0.0242 (7)	
C8	0.2402 (2)	0.0709 (3)	0.5542 (3)	0.0303 (8)	
H8A	0.2858	0.0244	0.6088	0.036*	
C9	0.1776 (2)	0.1094 (3)	0.5760 (3)	0.0338 (8)	
H9A	0.1805	0.0906	0.6466	0.041*	
C10	0.1096 (2)	0.1765 (3)	0.4932 (3)	0.0297 (8)	
H10A	0.0665	0.2014	0.5091	0.036*	
C11	0.16579 (19)	0.1707 (2)	0.3712 (3)	0.0219 (7)	
C12	0.16033 (19)	0.2076 (2)	0.2646 (3)	0.0209 (7)	
C13	0.0615 (9)	0.8631 (8)	0.2607 (13)	0.082 (2)	0.50
H13A	0.0782	0.9287	0.2404	0.098*	0.50
H13B	0.1113	0.8448	0.3432	0.098*	0.50
C14	−0.0247 (9)	0.8802 (6)	0.2597 (14)	0.082 (2)	0.50
H14A	−0.0759	0.8875	0.1739	0.098*	0.50
C15	−0.0222 (8)	0.9778 (6)	0.3227 (11)	0.082 (2)	0.50
H15A	−0.0019	1.0347	0.2950	0.123*	0.50
H15B	0.0202	0.9693	0.4093	0.123*	0.50
H15C	−0.0832	0.9925	0.3046	0.123*	0.50

### Atomic displacement parameters (Å^2^)

**Table:**

	*U*^11^	*U*^22^	*U*^33^	*U*^12^	*U*^13^	*U*^23^
Zn1	0.0229 (3)	0.0194 (3)	0.0300 (3)	0.000	0.0193 (2)	0.000
S1	0.0167 (8)	0.0184 (7)	0.0179 (19)	−0.0003 (7)	0.0114 (10)	0.0012 (7)
O1	0.0256 (9)	0.0259 (17)	0.0295 (12)	0.0019 (12)	0.0175 (9)	0.0001 (12)
O2	0.0256 (9)	0.0259 (17)	0.0295 (12)	0.0019 (12)	0.0175 (9)	0.0001 (12)
O3	0.0256 (9)	0.0259 (17)	0.0295 (12)	0.0019 (12)	0.0175 (9)	0.0001 (12)
O4	0.0256 (9)	0.0259 (17)	0.0295 (12)	0.0019 (12)	0.0175 (9)	0.0001 (12)
O5	0.077 (4)	0.033 (3)	0.070 (3)	−0.001 (3)	0.067 (3)	−0.004 (3)
O6	0.077 (4)	0.033 (3)	0.070 (3)	−0.001 (3)	0.067 (3)	−0.004 (3)
N1	0.0208 (12)	0.0242 (14)	0.0252 (14)	0.0010 (11)	0.0150 (11)	0.0031 (11)
N2	0.0233 (13)	0.0260 (15)	0.0242 (14)	−0.0001 (11)	0.0162 (11)	−0.0012 (11)
C1	0.0269 (16)	0.0261 (18)	0.0290 (18)	0.0022 (14)	0.0176 (14)	0.0056 (14)
C2	0.0328 (17)	0.034 (2)	0.0276 (18)	0.0024 (15)	0.0223 (15)	0.0040 (15)
C3	0.0285 (17)	0.036 (2)	0.0334 (19)	0.0027 (15)	0.0226 (15)	−0.0001 (15)
C4	0.0247 (15)	0.0268 (18)	0.0260 (17)	0.0018 (13)	0.0159 (14)	−0.0026 (14)
C5	0.0256 (15)	0.0322 (19)	0.0317 (19)	0.0062 (14)	0.0183 (14)	−0.0049 (15)
C6	0.0281 (16)	0.0296 (19)	0.0315 (19)	0.0078 (14)	0.0171 (15)	0.0004 (14)
C7	0.0252 (15)	0.0253 (17)	0.0220 (16)	−0.0016 (13)	0.0129 (13)	−0.0025 (13)
C8	0.0309 (17)	0.031 (2)	0.0263 (18)	0.0050 (14)	0.0140 (15)	0.0049 (14)
C9	0.0384 (18)	0.044 (2)	0.0231 (18)	0.0017 (17)	0.0198 (16)	0.0039 (16)
C10	0.0302 (16)	0.038 (2)	0.0285 (19)	0.0045 (15)	0.0208 (15)	0.0007 (15)
C11	0.0248 (15)	0.0199 (16)	0.0251 (17)	−0.0014 (13)	0.0163 (14)	−0.0029 (13)
C12	0.0198 (14)	0.0196 (16)	0.0259 (17)	−0.0012 (13)	0.0141 (13)	−0.0024 (13)
C13	0.134 (8)	0.031 (3)	0.146 (6)	−0.012 (3)	0.119 (6)	−0.008 (4)
C14	0.134 (8)	0.031 (3)	0.146 (6)	−0.012 (3)	0.119 (6)	−0.008 (4)
C15	0.134 (8)	0.031 (3)	0.146 (6)	−0.012 (3)	0.119 (6)	−0.008 (4)

### Geometric parameters (Å, º)

**Table:**

Zn1—O1	1.959 (4)	N2—C11	1.358 (4)
Zn1—O1^i^	1.959 (4)	C1—C2	1.402 (4)
Zn1—N2^i^	2.123 (3)	C1—H1A	0.9400
Zn1—N2	2.123 (3)	C2—C3	1.362 (5)
Zn1—N1	2.142 (2)	C2—H2A	0.9400
Zn1—N1^i^	2.142 (2)	C3—C4	1.408 (4)
S1—O1^i^	1.298 (5)	C3—H3A	0.9400
S1—O4^i^	1.355 (11)	C4—C12	1.408 (4)
S1—O3	1.446 (11)	C4—C5	1.424 (4)
S1—O2	1.459 (4)	C5—C6	1.348 (5)
S1—O4	1.492 (12)	C5—H5A	0.9400
S1—O1	1.505 (4)	C6—C7	1.440 (4)
S1—O3^i^	1.529 (12)	C6—H6A	0.9400
S1—O2^i^	1.998 (4)	C7—C11	1.396 (4)
O1—S1^i^	1.298 (4)	C7—C8	1.407 (4)
O1—O2^i^	1.435 (6)	C8—C9	1.362 (4)
O2—O1^i^	1.435 (6)	C8—H8A	0.9400
O2—S1^i^	1.998 (4)	C9—C10	1.389 (5)
O3—O4^i^	0.223 (14)	C9—H9A	0.9400
O3—S1^i^	1.529 (12)	C10—H10A	0.9400
O4—O3^i^	0.223 (14)	C11—C12	1.441 (4)
O4—S1^i^	1.355 (11)	C13—C14	1.508 (8)
O5—C13	1.394 (9)	C13—H13A	0.9800
O5—H5	0.8300	C13—H13B	0.9800
O6—C14	1.380 (8)	C14—C15	1.499 (9)
O6—H6	0.8300	C14—H14A	0.9900
N1—C1	1.324 (4)	C15—H15A	0.9700
N1—C12	1.357 (4)	C15—H15B	0.9700
N2—C10	1.329 (4)	C15—H15C	0.9700
			
O1—Zn1—N2^i^	137.27 (13)	C10—N2—C11	117.7 (3)
O1^i^—Zn1—N2^i^	110.38 (13)	C10—N2—Zn1	129.0 (2)
O1—Zn1—N2	110.38 (13)	C11—N2—Zn1	113.3 (2)
O1^i^—Zn1—N2	137.27 (13)	N1—C1—C2	122.7 (3)
N2^i^—Zn1—N2	110.75 (14)	N1—C1—H1A	118.6
O1—Zn1—N1	106.45 (13)	C2—C1—H1A	118.6
O1^i^—Zn1—N1	88.75 (13)	C3—C2—C1	119.3 (3)
N2^i^—Zn1—N1	92.67 (9)	C3—C2—H2A	120.3
N2—Zn1—N1	78.32 (9)	C1—C2—H2A	120.3
O1—Zn1—N1^i^	88.75 (13)	C2—C3—C4	120.0 (3)
O1^i^—Zn1—N1^i^	106.45 (13)	C2—C3—H3A	120.0
N2^i^—Zn1—N1^i^	78.32 (9)	C4—C3—H3A	120.0
N2—Zn1—N1^i^	92.67 (9)	C3—C4—C12	116.6 (3)
N1—Zn1—N1^i^	164.24 (14)	C3—C4—C5	123.7 (3)
O1^i^—S1—O4^i^	131.8 (5)	C12—C4—C5	119.6 (3)
O1^i^—S1—O3	139.5 (5)	C6—C5—C4	121.2 (3)
O4^i^—S1—O2	105.4 (5)	C6—C5—H5A	119.4
O3—S1—O2	111.2 (5)	C4—C5—H5A	119.4
O1^i^—S1—O4	109.5 (5)	C5—C6—C7	120.7 (3)
O4^i^—S1—O4	118.1 (7)	C5—C6—H6A	119.6
O3—S1—O4	109.9 (3)	C7—C6—H6A	119.6
O2—S1—O4	110.2 (5)	C11—C7—C8	117.8 (3)
O4^i^—S1—O1	105.6 (6)	C11—C7—C6	119.5 (3)
O3—S1—O1	108.2 (6)	C8—C7—C6	122.7 (3)
O2—S1—O1	109.3 (2)	C9—C8—C7	119.0 (3)
O4—S1—O1	107.9 (3)	C9—C8—H8A	120.5
O1^i^—S1—O3^i^	115.3 (5)	C7—C8—H8A	120.5
O4^i^—S1—O3^i^	112.9 (3)	C8—C9—C10	119.7 (3)
O3—S1—O3^i^	104.9 (7)	C8—C9—H9A	120.2
O2—S1—O3^i^	107.0 (5)	C10—C9—H9A	120.2
O1—S1—O3^i^	116.1 (3)	N2—C10—C9	123.1 (3)
O1^i^—S1—O2^i^	91.9 (2)	N2—C10—H10A	118.5
O4^i^—S1—O2^i^	90.3 (5)	C9—C10—H10A	118.5
O3—S1—O2^i^	86.9 (5)	N2—C11—C7	122.8 (3)
O2—S1—O2^i^	154.2 (3)	N2—C11—C12	117.5 (3)
O4—S1—O2^i^	78.4 (4)	C7—C11—C12	119.7 (3)
O3^i^—S1—O2^i^	84.5 (4)	N1—C12—C4	123.3 (3)
O1^i^—O1—S1^i^	74.3 (2)	N1—C12—C11	117.4 (2)
O1^i^—O1—O2^i^	134.1 (3)	C4—C12—C11	119.2 (3)
S1^i^—O1—O2^i^	64.3 (3)	O5—C13—C14	115.9 (14)
O1^i^—O1—S1	56.15 (18)	O5—C13—H13A	108.3
O2^i^—O1—S1	85.6 (3)	C14—C13—H13A	108.3
O1^i^—O1—Zn1	72.35 (12)	O5—C13—H13B	108.3
S1^i^—O1—Zn1	146.1 (3)	C14—C13—H13B	108.3
O2^i^—O1—Zn1	142.0 (3)	H13A—C13—H13B	107.4
S1—O1—Zn1	128.1 (2)	O6—C14—C15	109.9 (13)
O1^i^—O2—S1	53.3 (2)	O6—C14—C13	112.9 (16)
O1^i^—O2—S1^i^	48.7 (2)	C15—C14—C13	113.1 (8)
O4^i^—O3—S1	62 (5)	O6—C14—H14A	106.9
O4^i^—O3—S1^i^	76 (6)	C15—C14—H14A	106.9
O3^i^—O4—S1^i^	110 (5)	C13—C14—H14A	106.9
O3^i^—O4—S1	95 (6)	C14—C15—H15A	109.5
C13—O5—H5	109.5	C14—C15—H15B	109.5
C14—O6—H6	109.5	H15A—C15—H15B	109.5
C1—N1—C12	118.1 (2)	C14—C15—H15C	109.5
C1—N1—Zn1	129.2 (2)	H15A—C15—H15C	109.5
C12—N1—Zn1	112.58 (19)	H15B—C15—H15C	109.5
			
S1^i^—S1—O1—O1^i^	144.5 (10)	O2^i^—S1—O3—S1^i^	−14.0 (4)
O4^i^—S1—O1—O1^i^	−132.2 (5)	S1^i^—S1—O4—O3^i^	132 (6)
O3—S1—O1—O1^i^	−140.5 (5)	O1^i^—S1—O4—O3^i^	−135 (6)
O2—S1—O1—O1^i^	−19.2 (4)	O4^i^—S1—O4—O3^i^	53 (6)
O4—S1—O1—O1^i^	100.7 (6)	O3—S1—O4—O3^i^	54 (7)
O3^i^—S1—O1—O1^i^	101.9 (7)	O2—S1—O4—O3^i^	−69 (6)
O2^i^—S1—O1—O1^i^	153.1 (5)	O1—S1—O4—O3^i^	172 (6)
O1^i^—S1—O1—S1^i^	−144.5 (10)	O2^i^—S1—O4—O3^i^	137 (6)
O4^i^—S1—O1—S1^i^	83.3 (9)	O1^i^—S1—O4—S1^i^	92.8 (5)
O3—S1—O1—S1^i^	75.1 (8)	O4^i^—S1—O4—S1^i^	−79.2 (8)
O2—S1—O1—S1^i^	−163.7 (10)	O3—S1—O4—S1^i^	−77.5 (7)
O4—S1—O1—S1^i^	−43.8 (8)	O2—S1—O4—S1^i^	159.6 (5)
O3^i^—S1—O1—S1^i^	−42.6 (8)	O1—S1—O4—S1^i^	40.3 (5)
O2^i^—S1—O1—S1^i^	8.7 (8)	O3^i^—S1—O4—S1^i^	−132 (6)
S1^i^—S1—O1—O2^i^	−8.7 (8)	O2^i^—S1—O4—S1^i^	4.9 (4)
O1^i^—S1—O1—O2^i^	−153.1 (5)	O1—Zn1—N1—C1	−68.4 (3)
O4^i^—S1—O1—O2^i^	74.7 (4)	O1^i^—Zn1—N1—C1	−37.4 (3)
O3—S1—O1—O2^i^	66.4 (4)	N2^i^—Zn1—N1—C1	72.9 (3)
O2—S1—O1—O2^i^	−172.3 (2)	N2—Zn1—N1—C1	−176.4 (3)
O4—S1—O1—O2^i^	−52.5 (6)	N1^i^—Zn1—N1—C1	127.4 (3)
O3^i^—S1—O1—O2^i^	−51.2 (6)	O1—Zn1—N1—C12	116.5 (2)
S1^i^—S1—O1—Zn1	152.2 (8)	O1^i^—Zn1—N1—C12	147.5 (2)
O1^i^—S1—O1—Zn1	7.8 (2)	N2^i^—Zn1—N1—C12	−102.2 (2)
O4^i^—S1—O1—Zn1	−124.4 (4)	N2—Zn1—N1—C12	8.5 (2)
O3—S1—O1—Zn1	−132.7 (4)	N1^i^—Zn1—N1—C12	−47.68 (19)
O2—S1—O1—Zn1	−11.4 (5)	O1—Zn1—N2—C10	72.9 (3)
O4—S1—O1—Zn1	108.4 (6)	O1^i^—Zn1—N2—C10	101.2 (3)
O3^i^—S1—O1—Zn1	109.7 (6)	N2^i^—Zn1—N2—C10	−95.3 (3)
O2^i^—S1—O1—Zn1	160.9 (4)	N1—Zn1—N2—C10	176.3 (3)
N2^i^—Zn1—O1—O1^i^	−50.2 (4)	N1^i^—Zn1—N2—C10	−16.8 (3)
N2—Zn1—O1—O1^i^	146.2 (3)	O1—Zn1—N2—C11	−111.1 (2)
N1—Zn1—O1—O1^i^	62.8 (4)	O1^i^—Zn1—N2—C11	−82.9 (3)
N1^i^—Zn1—O1—O1^i^	−121.4 (4)	N2^i^—Zn1—N2—C11	80.7 (2)
O1^i^—Zn1—O1—S1^i^	11.1 (3)	N1—Zn1—N2—C11	−7.7 (2)
N2^i^—Zn1—O1—S1^i^	−39.1 (6)	N1^i^—Zn1—N2—C11	159.2 (2)
N2—Zn1—O1—S1^i^	157.3 (5)	C12—N1—C1—C2	0.8 (5)
N1—Zn1—O1—S1^i^	73.9 (5)	Zn1—N1—C1—C2	−174.1 (2)
N1^i^—Zn1—O1—S1^i^	−110.3 (5)	N1—C1—C2—C3	0.3 (5)
O1^i^—Zn1—O1—O2^i^	141.2 (8)	C1—C2—C3—C4	−1.3 (5)
N2^i^—Zn1—O1—O2^i^	91.0 (5)	C2—C3—C4—C12	1.2 (5)
N2—Zn1—O1—O2^i^	−72.6 (5)	C2—C3—C4—C5	−176.9 (3)
N1—Zn1—O1—O2^i^	−155.9 (5)	C3—C4—C5—C6	177.1 (3)
N1^i^—Zn1—O1—O2^i^	19.8 (5)	C12—C4—C5—C6	−1.0 (5)
O1^i^—Zn1—O1—S1	−6.76 (18)	C4—C5—C6—C7	−0.1 (5)
N2^i^—Zn1—O1—S1	−56.9 (4)	C5—C6—C7—C11	1.0 (5)
N2—Zn1—O1—S1	139.5 (3)	C5—C6—C7—C8	−177.5 (3)
N1—Zn1—O1—S1	56.1 (3)	C11—C7—C8—C9	−0.3 (5)
N1^i^—Zn1—O1—S1	−128.2 (3)	C6—C7—C8—C9	178.2 (3)
S1^i^—S1—O2—O1^i^	−30 (2)	C7—C8—C9—C10	1.2 (5)
O4^i^—S1—O2—O1^i^	129.5 (6)	C11—N2—C10—C9	−0.2 (5)
O3—S1—O2—O1^i^	135.8 (6)	Zn1—N2—C10—C9	175.6 (2)
O4—S1—O2—O1^i^	−102.0 (4)	C8—C9—C10—N2	−1.0 (5)
O1—S1—O2—O1^i^	16.4 (4)	C10—N2—C11—C7	1.2 (4)
O3^i^—S1—O2—O1^i^	−110.1 (4)	Zn1—N2—C11—C7	−175.3 (2)
O2^i^—S1—O2—O1^i^	3.7 (5)	C10—N2—C11—C12	−177.5 (3)
O1^i^—S1—O2—S1^i^	30 (2)	Zn1—N2—C11—C12	6.1 (3)
O4^i^—S1—O2—S1^i^	159 (2)	C8—C7—C11—N2	−0.9 (5)
O3—S1—O2—S1^i^	165 (2)	C6—C7—C11—N2	−179.5 (3)
O4—S1—O2—S1^i^	−72 (2)	C8—C7—C11—C12	177.7 (3)
O1—S1—O2—S1^i^	46 (2)	C6—C7—C11—C12	−0.9 (4)
O3^i^—S1—O2—S1^i^	−81 (2)	C1—N1—C12—C4	−0.8 (4)
O2^i^—S1—O2—S1^i^	33.3 (16)	Zn1—N1—C12—C4	174.9 (2)
S1^i^—S1—O3—O4^i^	128 (7)	C1—N1—C12—C11	176.2 (3)
O1^i^—S1—O3—O4^i^	25 (8)	Zn1—N1—C12—C11	−8.1 (3)
O2—S1—O3—O4^i^	−47 (7)	C3—C4—C12—N1	−0.2 (5)
O4—S1—O3—O4^i^	−169 (7)	C5—C4—C12—N1	178.0 (3)
O1—S1—O3—O4^i^	73 (7)	C3—C4—C12—C11	−177.1 (3)
O3^i^—S1—O3—O4^i^	−162 (7)	C5—C4—C12—C11	1.1 (4)
O2^i^—S1—O3—O4^i^	114 (7)	N2—C11—C12—N1	1.5 (4)
O1^i^—S1—O3—S1^i^	−103.3 (8)	C7—C11—C12—N1	−177.3 (3)
O4^i^—S1—O3—S1^i^	−128 (7)	N2—C11—C12—C4	178.6 (3)
O2—S1—O3—S1^i^	−175.2 (6)	C7—C11—C12—C4	−0.1 (4)
O4—S1—O3—S1^i^	62.4 (7)	O5—C13—C14—O6	67.9 (12)
O1—S1—O3—S1^i^	−55.1 (4)	O5—C13—C14—C15	−166.6 (14)
O3^i^—S1—O3—S1^i^	69.5 (5)		

Symmetry code: (i) −*x*, *y*, −*z*+1/2.

### Hydrogen-bond geometry (Å, º)

**Table:**

*D*—H···*A*	*D*—H	H···*A*	*D*···*A*	*D*—H···*A*
O5—H5···O3	0.83	2.00	2.74 (3)	149
O6—H6···O4	0.83	2.11	2.89 (2)	155

## References

[bb1] Batten, S. R. & Robson, R. (1998). *Chem. Commun.* pp. 1067–1068.

[bb2] Cui, J.-D., Zhong, K.-L. & Liu, Y.-Y. (2010). *Acta Cryst.* E**66**, m564. 10.1107/S1600536810014194 PMC2979185 21579046

[bb3] Jacobson, R. (1998). *REQAB.* Molecular Structure Corporation, The Woodlands, Texas, USA.

[bb4] Rigaku (2007). *CrystalClear* Rigaku Corporation, Tokyo, Japan.

[bb5] Sheldrick, G. M. (2008). *Acta Cryst.* A**64**, 112–122. 10.1107/S0108767307043930 18156677

[bb6] Zhang, L.-P., Ma, J.-F., Yang, J., Pang, Y.-Y. & Ma, J.-C. (2010). *Inorg. Chem.* **49**, 1535–1550. 10.1021/ic9019553 20095627

[bb7] Zhong, K.-L. (2010). *Acta Cryst.* E**66**, m131. 10.1107/S1600536809055433 PMC2979970 21579615

[bb8] Zhong, K.-L., Chen, L. & Chen, L. (2011). *Acta Cryst.* C**67**, m62–m64. 10.1107/S0108270110052558 21285499

[bb9] Zhu, Y.-M., Zhong, K.-L. & Lu, W.-J. (2006). *Acta Cryst.* E**62**, m2725–m2726.

